# Effect of Prey Species and Prey Densities on the Performance of Adult *Coenosia attenuata*

**DOI:** 10.3390/insects12080669

**Published:** 2021-07-23

**Authors:** Deyu Zou, Thomas A. Coudron, Lisheng Zhang, Weihong Xu, Jingyang Xu, Mengqing Wang, Xuezhuang Xiao, Huihui Wu

**Affiliations:** 1 Biological Control of Insects Research Laboratory, Institute of Plant Protection, Tianjin Academy of Agricultural Sciences, Tianjin 300384, China; zdyqiuzhen@126.com (D.Z.); xwh310@163.com (W.X.); jingyang_x@163.com (J.X.); 2 Biological Control of Insects Research Laboratory, USDA–Agricultural Research Service, Columbia, MO 65203, USA; coudront@missouri.edu; 3 Key Laboratory of Integrated Pest Management in Crops, Ministry of Agriculture, Institute of Plant Protection, Chinese Academy of Agricultural Sciences, Beijing 100193, China; zhanglisheng@caas.cn (L.Z.); wangmengqing@caas.cn (M.W.); 4 College of Horticulture and Landscape, Tianjin Agricultural University, Tianjin 300392, China; xxz24250@163.com

**Keywords:** *Coenosia attenuata*, mass rearing, wing damage, *Bradysia impatiens*, *Drosophila melanogaster*, fecundity

## Abstract

**Simple Summary:**

The predaceous fly *Coenosia attenuata* Stein has received attention because of its ability to effectively suppress a wide range of agricultural pests, such as fungus gnats, whiteflies and leaf miners. An effective level of control requires large numbers of *C. attenuata* to be available at low cost for release. Adult fungus gnats and drosophilids are now the main prey used to rear *C. attenuata* adults. However, previous studies showed *C. attenuata* fertility is lower when fed drosophilids compared to fungus gnats. The current study investigated the performance of *C. attenuata* adults when reared on different densities of adult *Drosophila melanogaster* Meigen or *Bradysia impatiens* (Johannsem). Results showed that the optimal prey density in the mass rearing of adult *C. attenuata* was 12–24 adult *B. impatiens* daily per predator. Additionally, *C. attenuata* adults suffered more wing damage, at some of the prey densities, when reared on *D. melanogaster* compared to *B. impatiens*. This information will be used to optimize rearing methods and decrease the cost of mass rearing in *C. attenuata*.

**Abstract:**

Mass production of *Coenosia attenuata* Stein at low cost is very important for their use as a biological control agent. The present study reports the performance of *C. attenuata* adults when reared on *Drosophila melanogaster* Meigen or *Bradysia impatiens* (Johannsem). Different densities (6, 9, 15, 24 and 36 adults per predator) of *D. melanogaster* or (6, 12, 24, 36 and 48 adults per predator) of *B. impatiens* were used at 26 ± 1 °C, 14:10 (L:D) and 70 ± 5% RH. The results concluded that *C. attenuata* adults had higher fecundity, longer longevity and less wing damage when reared on *B. impatiens* adults compared to *D. melanogaster* adults. Additionally, *C. attenuata* adults demonstrated greater difficulty catching and carrying heavier *D. melanogaster* adults than lighter *B. impatiens* adults. In this case, 12 to 24 adults of *B. impatiens* daily per predator were considered optimal prey density in the mass rearing of adult *C. attenuata*.

## 1. Introduction

The predaceous fly *Coenosia attenuata* Stein (Diptera: Muscidae), also known as “tiger fly”, “killer fly” or “hunter fly” [1,2,3,4], is native to Southern Europe [4,5] and has been reported to have spontaneously colonized a number of crops outdoors and in greenhouses in many countries worldwide [5,6,7,8,9,10,11,12,13,14,15,16,17,18,19,20,21]. It has received attention because of its ability to effectively suppress a wide range of agricultural pests, such as fungus gnats (Diptera: Sciaridae), whiteflies (Hemiptera: Aleyrodidae), leaf miners (Diptera: Agromyzidae), winged aphids (Hemiptera: Aphididae), leafhoppers of the genera *Eupteryx* (Hemiptera: Cicadellidae) and *Empoasca* (Hemiptera: Cicadellidae), midges (Diptera: Chironomidae), moth flies (Diptera: Psychodidae), shore flies (Diptera: Ephydridae) and fruit flies (Diptera: Drosophilidae) [7,8,13,22,23,24,25,26,27,28,29,30,31,32,33,34,35]. The wide range of prey used as food make the tiger-fly an attractive alternative to conventional control methods.

Intact wings play an important role in the life of *C. attenuata* adults. Adults of *C. attenuata* catch their prey while in flight and pursue targets at the range of 23–212 mm. Hence, they employ an interception strategy that is more energy efficient to intercept targets, which allows *C. attenuata* to cope with the extremely high line-of-sight rotation rates and thus prevents overcompensation of steering [36]. Adults of *C. attenuata* use mean flight speeds of 0.69 ms^−^^1^, mean wingbeat frequency of 306, 19 Hz and acceleration of mean peak 9.3 ms^−^^2^ to intercept prey [36]. The flight of *C. attenuata* individuals is affected by environmental factors, adjusting in response to changes in temperature, the number of prey flights and conspecific density [37]. Therefore, wing damage will cause negative effects on the life of *C. attenuata* adults.

Mass rearing of *C. attenuata* is important given the environmental, health and resistance issues associated with the use of chemical insecticides. To achieve an effective level of control, however, requires the production of a large number of *C. attenuata* at low cost. Adults of fungus gnat and drosophilid are now the main prey used to rear *C. attenuata* adults [17,23,38,39]. Rearing drosophilids is quick, easy and not particularly expensive. However, they were used primarily as a complement to the fungus gnat diet because *C. attenuata* fertility is lower when fed drosophilids compared to fungus gnats [23]. The reason why the performance of *C. attenuata* reared on drosophilids is lower than those reared on fungus gnats have not been assessed. The present study reports our finding that *C. attenuata* adults had less wing damage, higher fecundity and longer longevity when reared on *Bradysia impatiens* (Johannsem) (Diptera: Sciaridae) compared to *Drosophila melanogaster* Meigen (Diptera: Drosophilidae).

## 2. Materials and Methods

### 2.1. Vinegar Flies

The vinegar fly, *D. melanogaster* were reared on bananas in open plastic canisters (about 1200 cm^3^) in tissue bags (40 × 30 cm^2^, 0.4-mm mesh openings) closed with binder clips. Adults were introduced into the tissue bags and the adults of the following generation started to emerge after ca. 11 days. The colony was maintained in a laboratory incubator and held at 26 ± 1 °C, 14:10 (L:D) and 70 ± 5% RH.

### 2.2. Fungus Gnats

A colony of fungus gnats was initiated with about 400 *B. impatiens* adults captured from a greenhouse at Wuqing Experiment Station (Tianjin, China). Fungus gnats were reared using the method very similar to that reported by Zou et al. (2021) [39]. Modifications were made to simplify and improve the processes of rearing and collecting fungus gnats for use in bioassays. Briefly, 300 mL of black peat (Lvdimeijing Science and Technology Co., Ltd., Beijing, China) and 55 to 60 g of dry kidney bean powder were placed in an open plastic box (25.5 × 19 × 7.8 cm^3^). The mix was then moistened with 250 mL of tap water and 0.2-cm thick layer of moist coir (Shanghai Galuku Agricultural Science and Technology Co., Ltd. Shanghai, China; desalted, EC = 0.5, family pack, common grade) was placed on the top of the mix. Then the open plastic box was placed in a tissue bag (50 × 35 cm^2^, 0.4-mm mesh openings). In this case, 400 to 500 newly emerged adult fungus gnats were placed in the tissue bag and closed with a binder clip. Fresh rearing medium was prepared daily and new cultures were set up daily.

The new fungus gnat adults deposited eggs on the media consisted of black peat, tap water and kidney bean powder. Newly hatched larvae fed on the media and adults emerged after 18–22 d. The colony was maintained in a laboratory incubator and held at 26 ± 1 °C, 14:10 (L:D) and 70 ± 5% RH.

### 2.3. Tiger-Fly

The *C. attenuata* used to establish a laboratory colony in this study were collected at Leizhuangzi flower farm of Tianjin, China. Adults were provided an oviposition tissue cage (60 × 55 × 50 cm^3^), in which an open plastic box (29 × 20 × 7 cm^3^) containing black peat, tap water, kidney bean powder and eggs of *B. impatiens*. A 0.5-cm thick layer of moist coir was placed on the top of the rearing media and used for oviposition. *B. impatiens* and *D. melanogaster* adults were supplied as prey daily. Five to 6 days later, the plastic boxes containing eggs of *C. attenuata* and rearing media were removed to another cage and second and third instar larvae of *B. impatiens* were added to the box to feed larvae of *C. attenuata*. Distilled water was added to the box when the media became dry. About 20 to 21 days later, adults of *C. attenuata* emerged. The colony was maintained in an artificial climate chamber and held at 26 ± 1 °C, 14:10 (L:D) and 70 ± 5% RH.

### 2.4. Performance of Tiger-Fly Adults Reared on Different Prey Species at Different Prey Densities

Five female/male pairs of newly emerged adults of *C. attenuata* (<24-h-old), in the first generation that originated from field-caught adults, were transferred into tissue cages (60 × 55 × 50 cm^3^) containing an open 90-mm-diameter Petri dish containing of 0.7-cm thick layer of moist coir for oviposition. The moist coir was replenished after collecting egg daily. Two 110 cm strings were hung inside each cage and served as a perch for adult predators. In this case, 6, 9, 15, 24 and 36 adults of *D. melanogaster* and 6, 12, 24, 36 and 48 adults of *B. impatiens* (<24-h-old) were provided daily per predator adult. Prey were used only once and fresh prey were added daily. Five female/male pairs of *C. attenuata* adults (in one cage) were tested until death in each treatment and replicated 9 times. In total, 45 pairs of *C. attenuata* adults were tested in each treatment within a phytotron held at 26 ± 1 °C, 14:10 (L:D) and 70 ± 5% RH. Wing damage in *C. attenuata* adults was measured daily using a Mitutoyo 500-196-30 digital caliper (Mitutoyo, Kawasaki, Japan). Wing damage occurred along the long axis of wing. The extent of damage was calculated as a percentage using the length of damaged wing/the total length of wing × 100%. Both wings were assessed and the average was taken. The numbers of surviving and killed prey were recorded daily. Eggs deposited in the moist coir were collected and counted from each cage daily using a 00-sized paintbrush. They were then placed in 60-mm-diameter Petri dishes containing a single 55-mm-diameter filter paper, moistened with distilled water, sealed with Parafilm and inverted to keep the eggs moist. Hatch occurred ca. 6 days after oviposition and egg viability was calculated. Distilled water was sprayed to each tissue cage two times in the morning and afternoon per day. Tiger-flies were maintained in this manner until death.

### 2.5. Comparation of Body Weight and Body Length in C. attenuata, D. Melanogaster and B. Impatiens

The adults of *C. attenuata* caught prey in flight. So carrying prey with different body weights may cause different levels of wing damage for adults of *C. attenuata*. Adults of *C. attenuata* (<24-h-old) and *D. melanogaster* (<24-h-old) were placed in tissue bags (40 × 30 cm^2^) and held in a Siemens BCD-501W fridge (Siemens, Nanjing, China) at −20 °C for 2 min before taking body length and weight measurements. Adults of *B. impatiens* (<24-h-old) were handled in the same way with *C. attenuata* and *D. melanogaster* and held at −20 °C for 3 min before using. Adult body length was measured (in resting position) from the apex of the head to the wing tip for *C. attenuata* and *D. melanogaster*. For *B. impatiens*, body length was measured (in resting position) from the apex of the head to the abdomen tip. Body length measurements were made using a Mitutoyo 500-196-30 digital caliper (Mitutoyo, Kawasaki, Japan). Weight measurements were made using a Sartorius BP 211D (Sartorius AG, Göttingen, Germany) balance. In this case, 30 females and 30 males were measured for each species.

### 2.6. Statistical Analyses

One-way ANOVA with subsequent Tukey’s HSD test at α = 0.05 was used to compare the proportion of damaged wing, number of prey killed, preovipositional period, total fecundity between different prey densities, body weight and body length between insect species. To avoid possible mistakes due to multiple testing of the same data base, the *p*-values were Bonferroni corrected. Two sample t-tests for means were used to compare proportion of damaged wing, preovipositional period and total fecundity between prey species. These comparisons were carried out on day 4 and the last day. The proportion of eggs successfully hatched was compared between treatments by the Chi-square test at α = 0.01. All the statistical tests were carried out using SAS version 9.4.

## 3. Results

### 3.1. Comparison of Wing Damage of C. attenuata When Reared on Different Prey at Different Prey Densities

**Figure 1:**
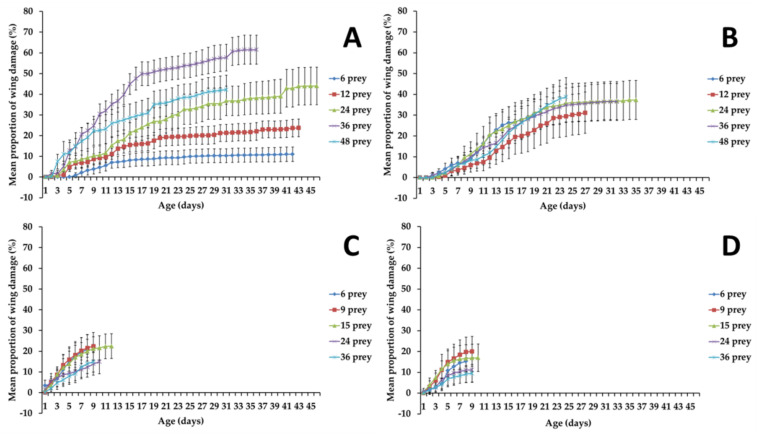
Mean proportion of damaged wings of all *Coenosia attenuata* when fed different prey at different densities daily per predator adult (mean values with 95% confidence intervals; error bars: 95% CI): (**A**) females fed 6, 12, 24, 36 and 48 adults of *Bradysia impatiens*; (**B**) males fed 6, 12, 24, 36 and 48 adults of *Bradysia impatiens*; (**C**) females fed 6, 9, 15, 24 and 36 adults of *Drosophila melanogaster*; (**D**) males fed 6, 9, 15, 24 and 36 adults of *Drosophila melanogaster*.

The mean proportion of damaged wings of *C. attenuata* females fed on *D. melanogaster* and males fed on *B. impatiens* or *D. melanogaster* did not differ significantly between prey densities at early ages (day 4) (*F*_4, 220_ = 1.27, *p* = 0.2826; *F*_4, 220_ = 1.55, *p* = 0.1886; *F*_4, 220_ = 2.4, *p* = 0.0513, respectively) (Figure 1B–D). However, *C. attenuata* females fed 48 adults of *B. impatiens* daily lost more wings compared with those fed 12 adults of *B. impatiens* daily on day 4 (*F*_1, 88_ = 9.39, *p* = 0.0029, Bonferroni-corrected *p* = 0.005) (Figure 1A). The wing damage increased with age in every case, but at different rates depending on prey species and density. When fed with *B. impatiens*, *C. attenuata* females showed significant differences at late age (31 days) between prey densities of 12 and 24 (*F*_1, 88_ = 13.15, *p* = 0.0004, Bonferroni-corrected *p* = 0.005), or between prey densities of 36 and 48 (*F*_1, 88_ = 10.16, *p* = 0.002, Bonferroni-corrected *p* = 0.005) (Figure 1A). For *C. attenuata* males, there were no significant differences between prey densities at late age (19 days) (*F*_4, 220_ = 0.63, *p* = 0.6425) (Figure 1B). When fed with *D. melanogaster*, both females and males of *C. coenosia* did not show significantly different wing damage between prey densities at later age (8 days) (*F*_4, 220_ = 2.08, *p* = 0.0848; *F*_4, 220_ = 2.07, *p* = 0.0856, respectively) (Figure 1C,D).

Females and males of *C. attenuata* had much shorter longevity when fed *D. melanogaster* adults compared to those fed *B. impatiens* adults (maximum age of 12 for *D. melanogaster* and of 46 for *B. impatiens* in females; maximum age of 10 for *D. melanogaster* and of 35 for *B. impatiens* in males). Mean proportion of wing damage in flies fed with *B. impatiens* at age 8 ranged from 3.25 to 22.42% in females and from 4.68 to 9.58% in males, while in flies fed with *D. melanogaster* it ranged from 12.28 to 21.56% in females and from 9.16 to 19.77% in males (Figure 1).

*C. attenuata* females fed 6 adults of *D. melanogaster* daily lost significantly more wings than those fed 6 adults of *B. impatiens* daily on day 4 (*t* = −2.935, *df* = 88, *p* = 0.0043) (Figure 1A,C). However, there was no significant difference in wing damage for *C. attenuata* females fed 24 adults of *D. melanogaster* compared with those fed 24 adults of *B. impatiens* daily, or for *C. attenuata* females fed 36 adults of *D. melanogaster* compared with those fed 36 adults of *B. impatiens* daily on day 4 (*t* = −1.934, *df* = 88, *p* = 0.0564; *t* = −0.447, *df* = 88, *p* = 0.6561, respectively) (Figure 1A,C). *C. attenuata* females lost significantly more wings when fed adults of *D. melanogaster* daily compared to *B. impatiens* at the prey density of 6 and 36 on day 8 (*t* = −4.556, *df* = 88, *p* < 0.0001; *t* = 2.803, *df* = 88, *p* = 0.0062, respectively). However, there was no significant difference in wing damage for *C. attenuata* females when fed 24 adults of *D. melanogaster* compared with those fed 24 adults of *B. impatiens* on day 8 (*t* = −0.874, *df* = 88, *p* = 0.3847) (Figure 1A,C).

There was no significant difference in wing damage for *C. attenuata* males fed 6 adults of *D. melanogaster* compared with those fed 6 adults of *B. impatiens*, or for *C. attenuata* males fed 36 adults of *D. melanogaster* compared with those fed 36 adults of *B. impatiens* on day 4 (*t* = −1.35, *df* = 88, *p* = 0.1805; *t* = −1.1, *df* = 88, *p* = 0.2743, respectively) (Figure 1B,D). *C. attenuata* males lost significantly more wings when fed adults of *D. melanogaster* daily compared to *B. impatiens* at the prey density of 24 on day 4 (*t* = −2.302, *df* = 88, *p* = 0.0237) and the prey density of 6 on day 8 (*t* = −2.631, *df* = 88, *p* = 0.01). However, there was no significant difference in wing damage for *C. attenuata* males when fed adults of *D. melanogaster* daily compared to *B. impatiens* at the prey density of 24 or 36 on day 8 (*t* = −0.362, *df* = 88, *p* = 0.7181; *t* = −0.482, *df* = 88, *p* = 0.6311, respectively) (Figure 1B,D).

### 3.2. Number of Prey killed by C. attenuata Reared on Different Prey at Different Prey Densities

**Figure 2:**
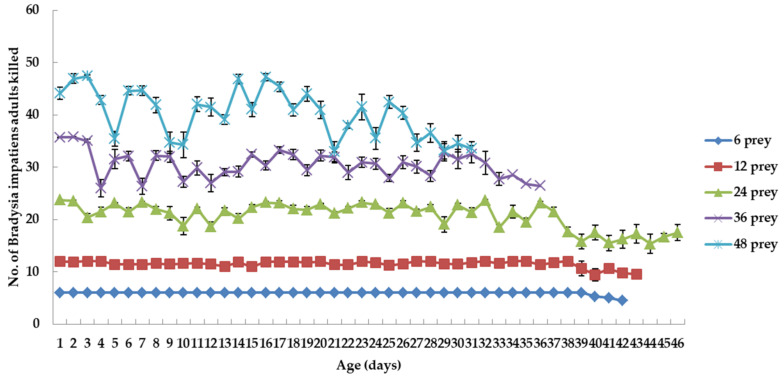
Mean number of prey killed per *C. attenuata* adult when fed 6, 12, 24, 36 and 48 adults of *B. impatiens* daily per predator adult (values are mean ± SE, single values were present when only one adult *C. attenuata* was left in the end).

*C. attenuata* adults killed all *B. impatiens* adults when fed 6 prey daily per predator adult except for the last 3 days, which suggests 6 adults of *B. impatiens* are not enough for *C. attenuata* adults (Figure 2). Most of *B. impatiens* adults were killed by *C. attenuata* adults when fed 12 prey daily per predator adult. The number of prey killed by *C. attenuata* adults fluctuated when fed 24, 36 and 48 prey daily per predator adult. The numbers of prey killed per predator daily were 15.33 to 23.77, 25.99 to 35.70 and 33.00 to 47.45 for *C. attenuata* adults fed 24, 36 and 48 prey daily per predator adult, respectively. The number of prey killed daily per predator decreased in the last few days with the increase of age and the mean proportion of damaged wings for *C. attenuata* adults fed 12, 24, 36 and 48 prey daily per predator adult (Figure 2). The number of *B. impatiens* adults killed daily per predator adult differed significantly between prey densities at early ages (day 4) (*p* < 0.0001 for 9 comparisons), except for densities of 24 vs. 36 (*F*_1, 16_ = 5.61, *p* = 0.0308, Bonferroni-corrected *p* = 0.005) (Figure 2). At later age (day 31), the number of *B. impatiens* adults killed daily per predator adult differed significantly between prey densities (*p* < 0.0001 for 9 comparisons), except for densities of 36 vs. 48 (*F*_1, 4_ = 0.26, *p* = 0.6392, Bonferroni-corrected *p* = 0.005) (Figure 2).

**Figure 3:**
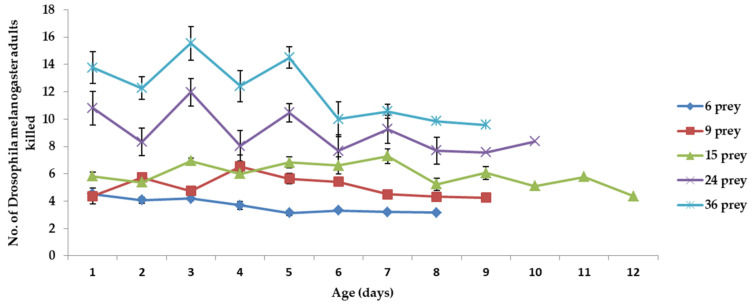
Mean number of prey killed per *C. attenuata* adult when fed 6, 9, 15, 24 and 36 adults of *D. melanogaster* daily per predator adult (values are mean ± SE, single values were present when only one adult *C. attenuata* was left in the end).

*C. attenuata* adults killed 3.17 to 4.52 *D. melanogaster* adults when fed 6 prey adults daily per predator adult (Figure 3). The number of prey killed by *C. attenuata* adults fluctuated when fed 9, 15, 24 and 36 prey daily per predator adult. The numbers of prey killed daily per predator were 4.27 to 6.54, 4.37 to 7.29, 7.57 to 11.98 and 9.58 to 15.55 for *C. attenuata* adults fed 9, 15, 24 and 36 prey daily per predator adult, respectively. The number of prey killed per predator daily decreased in the last few days with the increase of age and the proportion of broken wings for *C. attenuata* adults in all treatments. The number of *D. melanogaster* adults killed daily per predator adult differed significantly with prey densities of 6 vs. 9 (*F*_1, 16_ = 10.64, *p* = 0.0049, Bonferroni-corrected *p* = 0.005), 9 vs. 36 (*F*_1, 16_ = 17.21, *p* = 0.0008, Bonferroni-corrected *p* = 0.005) and with the prey densities of 6 vs. 15, 6 vs. 24, 6 vs. 36 and 15 vs. 36 (all *p* < 0.0001) at early ages (day 4). No significant differences were found in the other 4 comparisons at early ages (day 4) (Figure 3). Additionally, no significant differences were found in all 10 comparisons at later age (day 8) (Figure 3).

### 3.3. Preovipositional Period of C. attenuata Female Reared on Different Prey at Different Prey Densities

**Figure 4:**
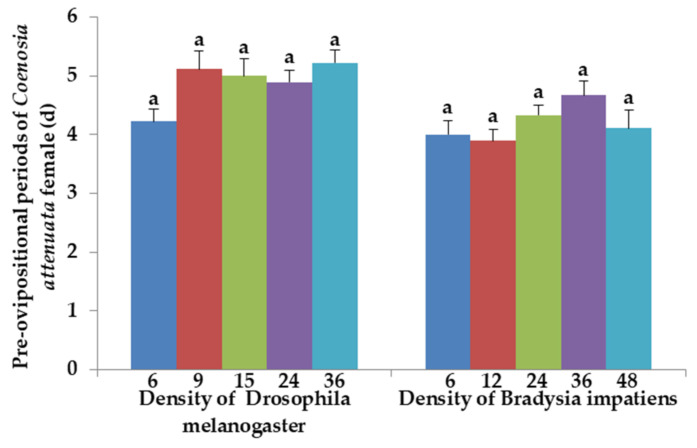
Preovipositional periods of *C. attenuata* female adult when fed 6, 9, 15, 24 and 36 adults of *D. melanogaster* and 6, 12, 24, 36 and 48 adults of *B. impatiens* daily per predator adult. Different letters above each bar indicate significant differences between prey densities using one-way ANOVA, Tukey’s HSD test (*p* = 0.05 and n = 9).

There were no significant differences in preovipositional period in any of the treatments of *C. attenuata* females when fed 6, 9, 15, 24 or 36 adults of *D. melanogaster* prey (*F*_4, 40_ = 2.43, *p* = 0.064) (Figure 4). The preovipositional period of *C. attenuata* female was from 4.22 to 5.22 days when fed *D. melanogaster* prey. Similar, there were no significant differences in preovipositional period in any of the treatments of *C. attenuata* females when fed 6, 12, 24, 36 or 48 adults of *B. impatiens* (*F*_4, 40_ = 1.73, *p* = 0.162). The preovipositional period of *C. attenuata* female was from 3.89 to 4.67 days when fed *B. impatiens* prey. There were no significant differences in the preovipositional period for *C. attenuata* females when fed 6 adults of *D. melanogaster* prey or 6 adults of *B. impatiens* prey, or for *C. attenuata* females when fed 36 adults of *D. melanogaster* prey or 36 adults of *B. impatiens* prey (*t* = −0.686, *df* = 16, *p* = 0.5025; *t* = −1.715, *df* = 16, *p* = 0.1056, respectively) (Figure 4). The preovipositional periods were significantly longer for *C. attenuata* females fed 24 adults of *D. melanogaster* prey than of *C. attenuata* females fed 24 adults of *B. impatiens* prey. These differences, although statistically significant, were small (*t* = −2.132, *df* = 16, *p* = 0.0489) (Figure 4).

### 3.4. Total Fecundity of C. attenuata Female Reared on Different Prey at Different Prey Densities

**Figure 5:**
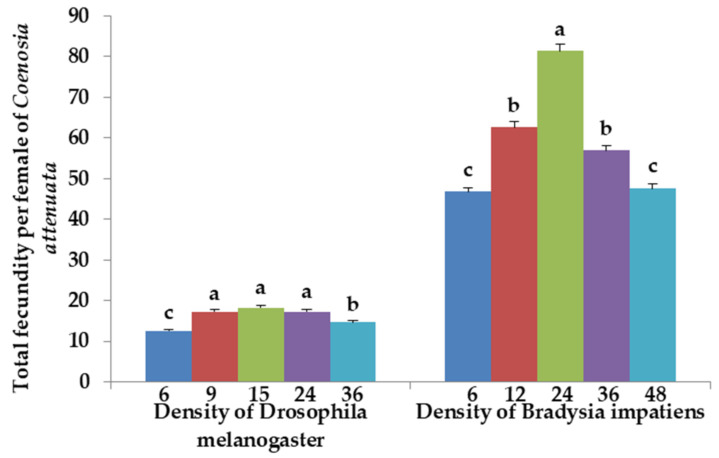
Total fecundity per female of *C. attenuata* when fed 6, 9, 15, 24 and 36 adults of *D. melanogaster* and 6, 12, 24, 36 and 48 adults of *B. impatiens* daily per predator adult. Different letters above each bar indicate significant differences between prey densities (corrected *p* value for multiple testing by Bonferroni correction is 0.005 and n = 9).

The total fecundity per female of *C. attenuata* fed *D. melanogaster* prey differed significantly with prey densities of 6 vs. 36 (*F*_1, 16_ = 17.38, *p* = 0.0007, Bonferroni-corrected *p* = 0.005), 9 vs. 36 (*F*_1, 16_ = 19.82, *p* = 0.0004, Bonferroni-corrected *p* = 0.005), 24 vs. 36 (*F*_1, 16_ = 18.90, *p* = 0.0005, Bonferroni-corrected *p* = 0.005) and the other 4 comparisons (6 vs. 9, 6 vs. 15, 6 vs. 24 and 15 vs. 36, all *p* < 0.0001). No significant differences were found in other 3 comparisons (Figure 5). The total fecundity per female of *C. attenuata* fed *B. impatiens* prey did not differ significantly with prey densities of 6 vs. 48 and 12 vs. 36 (*F*_1, 16_ = 0.17, *p* = 0.6829; *F*_1, 16_ = 9.54, *p* = 0.007, respectively, Bonferroni-corrected *p* = 0.005). However, the total fecundity per female of *C. attenuata* fed *B. impatiens* prey differed significantly between prey densities for the other 8 comparisons (all *p* < 0.0001) (Figure 5). Additionally, the total fecundity was much higher for females of *C. attenuata* fed *B. impatiens* adults than for those fed *D. melanogaster* prey at the same prey densities of 6, 24 and 36 (*t* = 31.971, *df* = 16, *p* < 0.0001; *t* = 36.609, *df* = 16, *p* < 0.0001; *t* = 32.954, *df* = 16, *p* < 0.0001, respectively).

### 3.5. Proportion of Eggs Successfully Hatched in C. attenuata Reared on Different Prey at Different Prey Densities

**Figure 6:**
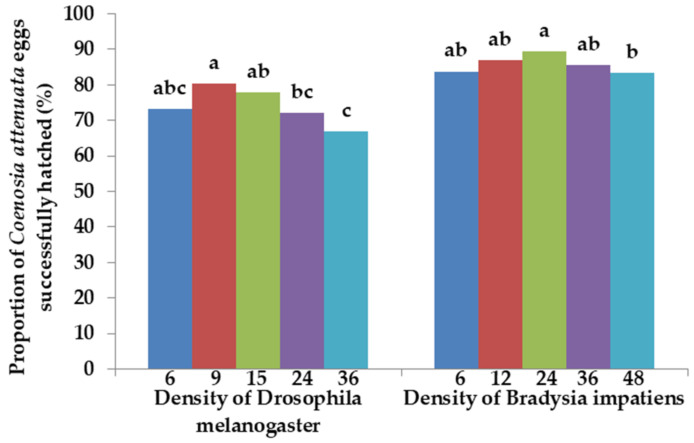
Proportion of eggs successfully hatched for *C. attenuata* when fed 6, 9, 15, 24 and 36 adults of *D. melanogaster* and 6, 12, 24, 36 and 48 adults of *B. impatiens* daily per predator adult. Different letters above each bar indicate significant differences between prey densities at a 0.01 level of significance using Chi-square test (n = 450, first 50 eggs were collected from each cage, 9 repetitions in each treatment).

There was a significantly higher proportion of eggs that successfully hatched for *C. attenuata* fed 9 adults of *D. melanogaster* than for those fed 24 and 36 adults of *D. melanogaster* daily per predator adult (χ^2^ = 8.37, *p* = 0.004; χ^2^ = 20.56, *p* < 0.0001, respectively) (Figure 6). However, there were no significant differences in the proportion of eggs that successfully hatched between *C. attenuata* fed 9 adults of *D. melanogaster* and those fed 6 or 15 adults of *D. melanogaster* (χ^2^ = 6.36, *p* = 0.012; χ^2^ = 0.81, *p* = 0.368, respectively). There were no significant differences in proportion of eggs successfully hatched between *C. attenuata* fed 24 adults of *B. impatiens* and those fed 6, 12 or 36 adults of *B. impatiens* (χ^2^ = 5.97, *p* = 0.015; χ^2^ = 1.28, *p* = 0.257; χ^2^ = 2.92, *p* = 0.087, respectively). The proportion of eggs that successfully hatched was significantly higher for *C. attenuata* fed 24 adults of *B. impatiens* than for those fed 48 adults of *B. impatiens* daily per predator adult (χ^2^ = 6.87, *p* = 0.009). Additionally, the proportion of eggs that successfully hatched was much higher for *C. attenuata* adults fed *B. impatiens* adults than for those fed *D. melanogaster* adults at the same prey densities of 6, 24 and 36 (χ^2^ = 15.14, *p* < 0.0001; χ^2^ = 43.35, *p* < 0.0001; χ^2^ = 43.26, *p* < 0.0001, respectively).

### 3.6. Comparation of Body Weight and Body Length in C. attenuata, D. melanogaster and B. impatiens

Adult females of *C. attenuata* were significantly longer than those of *D. melanogaster* and *B. impatiens* (*F*_2, 87_ = 1335.80, *p* < 0.0001) (Table 1). Adult females of *D. melanogaster* were significantly longer than those of *B. impatiens* (*F*_2, 87_ = 1335.80, *p* < 0.0001). Similar, adult males of *C. attenuata* were significantly longer than those of *D. melanogaster* and *B. impatiens* (*F*_2, 87_ = 2101.27, *p* < 0.0001) and the body length was significantly longer for adult males of *D. melanogaster* than for adult males of *B. impatiens* (*F*_2, 87_ = 2101.27, *p* < 0.0001).

Adult females of *C. attenuata* were significantly heavier than those of *D. melanogaster* and *B. impatiens* and there was a significant difference in the body weight of adult females between *D. melanogaster* and *B. impatiens* (*F*_2, 87_ = 2188.57, *p* < 0.0001). Similar, adult males of *C. attenuata* were significantly heavier than those of *D. melanogaster* and *B. impatiens* and there was a significant difference in the body weight of adult males between *D. melanogaster* and *B. impatiens* (*F*_2, 87_ = 1164.34, *p* < 0.0001) (Table 1).

## 4. Discussion

The flight of *C. attenuata* individuals was affected by environmental factors and was increased in response to increases in the number of prey flights [37]. Bonsignore (2016) found that predatory flights of adult *C. attenuata* comprised a small percentage (ca. 6%) of the total flights, with a predation success rate of 61% [37]. In our study, the mean proportion of damaged wings of *C. attenuata* females when fed 6, 12, 24 and 36 adults of *B. impatiens* daily per predator adult was increased in response to increases in the number of prey densities. However, the mean proportion of wing damage in *C. attenuata* females was lower for prey densities of 48 adults of *B. impatiens* than for prey densities of 36. The high density of 48 adults of *B. impatiens* probably increased the predation success rate and thereby decreased the mean proportion of damaged wings of *C. attenuata* female although the tiger-fly is regarded to have predation instinct [40,41]. Damaged wings of *C. attenuata* males fed on *B. impatiens* continued to increase with an increase in age of *C. attenuata*. However, the mean proportions of damaged wings in *C. attenuata* males were not consistent with those of females. Prey density did not cause significant effect on wing damage for *C. attenuata* males, which suggests prey density was not the only factor affecting wing damage in *C. attenuata* males. Being attacked by female *C. attenuata* and attempting to mate with female *C. attenuata* could also influence wing damage. Additionally, male adults required less prey compared to female adults, which means low prey densities could increase the predation success rate and thereby decreased the proportion of damaged wings of *C. attenuata* males. According to the damaged wings and longevity of *C. attenuata* adults, prey densities of 12 to 24 should be optimal density for mass rearing of adult *C. attenuata*.

Prey density of vinegar fly did not cause a significant effect on the mean proportion of damaged wings in both female and male adults of *C. attenuata*. It seems reasonable to conclude that the short lifespan of the tiger-fly was too short to manifest an effect of *D. melanogaster* prey density on wing damage of *C. attenuata* adults. *C. attenuata* adults fed *D. melanogaster* prey daily lost more wings compared to those fed *B. impatiens* prey at the same age for some prey density. This may be related to the increased difficulty in carrying heavier *D. melanogaster* adults than *B. impatiens* adults.

Bonsignore (2016) sorted adult *C. attenuata* flights into three groups, movement flights, territory defense flights and predatory flights in greenhouse [37]. However, we observed that there should be another type of flight, escape flights in cage. We hypothesize that adult *C. attenuata* want to escape from the cage when encountering high density of prey, resulting in less wing damage. Escaping from the environment with high prey density may be a self-protection response for adult *C. attenuata*.

We found that females lived longer than males, as reported by Kühne et al. (1997) [23]. However, these authors record 38 days and 33 days as the maximum female and male longevity, respectively, under laboratory conditions (25 °C and 50–60% RH) and an estimated longevity of eight weeks under greenhouse conditions. Predators fed *B. impatiens* adults in our study lived 46 days and 35 days as the maximum female and male longevity, respectively, possibly because they had a better food supply. However, in our study, predators fed adult *D. melanogaster* flies lived only 12 days and 10 days as the maximum female and male longevity, respectively. We speculate that *C. attenuata* adults were able to more easily capture lighter *B. impatiens* adults than heavier adult *D. melanogaster*. Additionally, adult *C. attenuata* were able to attack adult *B. impatiens* on the bottom of cage when they could only jump or crawl because of damaged wings. However, it is difficult for *C. attenuata* with damaged wings to capture adult *D. melanogaster*.

Female adult *C. attenuata* were found to exhibit a type I functional response to adult sciarid flies, which was conducted in glass vials 8 cm long and 8 cm in diameter at 25 °C at 60–80% RH, with a 16L:8D photoperiod. Sciarids were consumed in significantly different numbers at densities from 5 to 20 individuals (the number of killed flies changed from 2.90 to 8.4, respectively). However, increasing prey availability beyond 20 individuals resulted in no substantial increase in predation [30]. However, female adult *C. attenuata* were found to exhibit a type II functional response to adult *D. melanogaster* flies, which was conducted in Plexiglas cages with a dimension of 25 by 25 by 25 cm at 30 °C at 65 *±* 5% RH, with a 12L:12D photoperiod. *D. melanogaster* flies were consumed in significantly different numbers at densities from 5 to 55 individuals (the number of attacked flies changed from 3.50 to 5.67, respectively) [42]. Kühne (2000) states that each adult *C. attenuata* needs either 1.5 adults of *D. melanogaster* or 6.9 adults of *B. impatiens* per day [25]. We did not analyze the functional response to adult *B. impatiens* or *D. melanogaster* flies because more than one factor affected functional response, such as intraspecific competition and predation. The number of killed prey in our study was more than those mentioned above, which was probably caused by intraspecific competition and predation instinct resulting from cage and space differences. The flight ability of adult *B. impatiens* is weak and often some of them stayed on the bottom of cage which made it more convenient for adult *C. attenuata* without flight ability, because of damaged wings, to catch the adult *B. impatiens*. In contrast, the flight ability of adult *D. melanogaster* is strong and it is more difficult for adult *C. attenuata* with weakened flight ability to catch adult *D. melanogaster*, although adult *C. attenuata* has been proved to be more efficient in information sampling and processing than adult *D. melanogaster* [43,44].

The preoviposition period of *C. attenuata* is approximately 4 days [23]. Our reports showed similar preoviposition periods when fed adults of *B. impatiens* with 3.89–4.67 days and adults of *D. melanogaster* with 4.22–5.22 days. Prey density, prey species and damaged wings did not cause negative effects on the preoviposition period of *C. attenuata*. Sanderson et al. (2009) found the tiger-flies laid more eggs with fungus gnat prey than shore fly prey [27]. We found that the tiger-flies laid much more eggs with fungus gnat prey than vinegar fly prey. However, the total fecundity per female of *C. attenuata* did not continue to increase with an increase in prey density. Shorter life span probably cause the lower fecundity for *C. attenuata* female when fed adult *D. melanogaster* compared to adult *B. impatiens*. Martins et al. (2015) presented an optimized method for mass rearing *C. attenuata* with fungus gnats and Drosophilids as prey, where the number of adults that emerged per parental pair ranged from 1.8 to 9.0 (= per pair progeny production, or the number of adult offspring that emerged in each cage divided by the number of parental pairs) [38]. In our study, the number of adult offspring that emerged ranged from 4.96 to 7.64 and 21.16 to 39.27 at least for per parental pair when fed adult *D. melanogaster* and *B. impatiens*, respectively according to survival rates of larvae, percentages of pupation and adult emergence in our previous reports [17,39]. The proportion of eggs that successfully hatched was much higher for *C. attenuata* adults fed *B. impatiens* adults than for those fed *D. melanogaster* adults at the same prey densities. Longer longevity in male *C. attenuata* and lighter body weight in *B. impatiens* prey correlated to increased proportion of eggs that successfully hatched in *C. attenuata.*

Predation by adult *C. attenuata* is rapid, and adults take off as soon as they observe their prey in flight, although they do not know the absolute size of the potential prey prior to the flight [45]. One important physical factor affecting predator responses is prey size [46]. Body length and body weight of adult *C. attenuata*, *D. melanogaster* and *B. impatiens* were analyzed in our study to better understand the complexity of predation. We report body length and body weight of adult *C. attenuata* from Tianjin to be similar to those reported by us previously [17,39] and to those reported in Uruguay where the *C. attenuata* flies measured approximately 2.5–5.00 mm in length [47]. Body weight of adult *D. melanogaster* measured in this study is similar to those reported by Chen et al. (2019) [48]. Body length of adult *B. impatiens* analyzed in this study is similar to those reported by Wilkinson and Daugherty (1970) [49]. Obviously, it is more difficult for adult *C. attenuata* to catch and carry heavier adult *D. melanogaster* than lighter adult *B. impatiens*. Most importantly, it is more difficult for male adult *C. attenuata* to catch and carry female adult *D. melanogaster*, that are 74.15% weight of male adult of *C. attenuata* than to catch adult female *B. impatiens* that only weigh 28.57% of their weight.

In our study, we demonstrated that adult *B. impatiens* was an optimal prey in the mass rearing of adult *C. attenuata* although rearing drosophilids is quick, easy and not particularly expensive. In addition, we provide evidence that damage to wings of adult *C. attenuata* when fed adult *D. melanogaster* vs. *B. impatiens* is an important consideration for prey selection. We conclude a prey density of 12–24 adult fungus gnats daily per adult predator as optimal for mass rearing of adult *C. attenuata*. Rearing cost, nutritional difference, digestion efficiency, chemical, morphological and behavioral defense mechanisms of a prey will be explored in future studies.

## 5. Conclusions

We present the first report of wing damage for *C. attenuata* adults when reared on different prey. The results indicate that *C. attenuata* adults had higher fecundity, longer longevity and generally less wing damage when reared on *B. impatiens* compared to *D. melanogaster*. Lighter body weight and weaker flight ability in adult *B. impatiens* prey likely contributed to prolonged longevity and increased fecundity in adult *C. attenuata.* In this case, 12 to 24 adults of *B. impatiens* daily per predator were considered optimal prey density in the mass rearing of adult *C. attenuata* adult.

## Figures and Tables

**Table 1:** Body length (mm) and body weight (mg) of adult *Coenosia attenuata*, *Drosophila melanogaster* and *Bradysia impatiens* (n = 30).

Insects	Body Length of Adult Female ^a^ (mm)	Body Length of Adult Male ^a^ (mm)	Body Weight of Adult Female ^b^ (mg)	Body Weight of Adult Male ^b^ (mg)
*Coenosia attenuata*	4.49 ± 0.04 ^a^	3.68 ± 0.02 ^a^	2.52 ± 0.04 ^a^	1.47 ± 0.03 ^a^
*Drosophila melanogaster*	3.12 ± 0.02 ^b^	2.78 ± 0.02 ^b^	1.09 ± 0.01 ^b^	0.72 ± 0.01 ^b^
*Bradysia impatiens*	2.22 ± 0.02 ^c^	1.70 ± 0.02 ^c^	0.42 ± 0.01 ^c^	0.23 ± 0.01 ^c^

Values are mean ± SE. Means in columns with the same letter are not significantly different at a 0.05 level of significant. ^a^ Adult body length measured < 24 h after adult emergence. ^b^ Adult body weight measured < 24 h after adult emergence.

## Data Availability

Not applicable.
